# Repositioning a Displaced Right Ventricular Pacing Lead via Percutaneous Approach in Three Patients

**DOI:** 10.19102/icrm.2022.130604

**Published:** 2022-06-15

**Authors:** Ahmad Yaminisharif, Seyed Hossein Ahmadi Tafti, Ali Hosseinsabet, Akbar Shafiee

**Affiliations:** ^1^Tehran Heart Center, Cardiovascular Diseases Research Institute, Tehran University of Medical Sciences, Tehran, Iran

**Keywords:** Complication, lead perforation, permanent pacemaker, right ventricle

## Abstract

Lead-related complications compose a noticeable share of device-related complications. Pacemaker lead perforation is a recognized complication of lead implantation, particularly with active fixation leads, and should be considered in postoperative lead malfunction cases. We herewith present 3 challenging cases with ventricular pacemaker lead dispositioning who were successfully treated via percutaneous access.

## Introduction

The use of implantable cardiac devices has been accompanied by an increase in its complications.^1^ Lead-related complications comprise a noticeable share of device-related complications, and their management demands a high level of care and experience.^1^ The standard treatments for lead perforation are repositioning the lead by the reoperation of the generator pocket and placing its tip in the correct position.^2^ Percutaneous transcatheter repositioning of displaced passive leads in the coronary sinus and the right atrium (RA) has been performed before and was shown to be a safe and effective method.^3,4^ We herewith introduce 3 cases of displaced pacemaker leads treated successfully via percutaneous transcatheter traction and repositioning.

This study was approved by the ethics committee of Tehran Heart Center, and all cases in this study gave consent for anonymous publication of their data.

## Case presentations

### Case 1

An 88-year-old woman presented with a history of dual-chamber permanent pacemaker implantation 5 years ago following symptomatic episodes of complete heart block with progressive dyspnea on exertion. Pacemaker interrogation showed high right ventricular (RV) lead impedance (4,000 Ω) because of lead fracture. She was scheduled for the abandonment of this lead and the re-implantation of a new RV lead. Chest fluoroscopy demonstrated that the RA lead was floating in the RA. Because of the normal parameters of the RA lead, we decided not to change this lead. Left subclavian angiography revealed significant stenosis in the junction of the subclavian vein and the superior vena cava. We had to advance the new lead through a coronary sinus long sheath (CPS Direct SL II, Splittable Outer Guide Catheter; Abbott, Chicago, IL, USA). This active fixation lead was placed in the RV apex (CapSureFix model 4076; Medtronic, Minneapolis, MN, USA) and was attached to a new pulse generator. After 2 days, the patient suffered dysrhythmia, and an electrocardiogram revealed intermittent non-capture of the RV lead. Pacemaker interrogation confirmed the loss of sensing and pacing of this lead. On suspicion of RV lead perforation, transthoracic echocardiography was performed, which demonstrated perforation of the RV free wall and the pericardium by the active-fixation RV lead together with a small pericardial effusion without any signs of cardiac tamponade.

**Figure 1:**
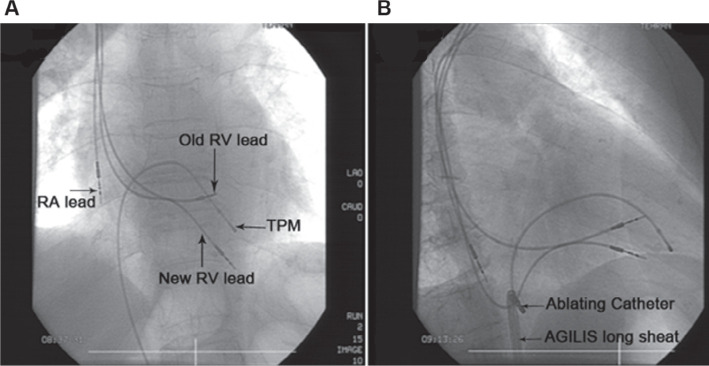
Chest fluoroscopy of patient 1. **A:** The tip of the new right ventricular lead seems to be in the pericardial space. **B:** Using an Agilis long sheath and ablating catheter, a hook was made, and the right ventricular lead was pulled back. *Abbreviations:* RA, right atrium; RV, right ventricle; TPM, trabeculae and papillary muscle.

Additionally, fluoroscopic images confirmed lead dislodgement **(Figure 1A)**. Due to the patient’s old age and reluctance to undergo surgery, we decided to perform percutaneous transcatheter repositioning of the displaced lead before considering surgical repositioning. First, via an Agilis long sheath (Agilis NxT, Steerable Introducer; Abbott), we advanced a deflectable ablating catheter from the left femoral vein to the RA and clasped the RA portion of the lead **(Figure 1B)**. Afterward, we pulled back the lead gently, withdrew the lead slowly, and repositioned it in the stable site of the RV apex under fluoroscopic guidance and without any complication. Simultaneous transthoracic echocardiography showed that the lead’s tip was lodged in the RV myocardium 3 mm from the pericardium and that the perforated site of the RV was sealed. The whole procedure was performed with a cardiac surgeon on standby and in a setting conducive to prompt pericardiocentesis. A postoperative pacing check demonstrated stable pacing and sensing thresholds in the RV lead. One day after the procedure, no change in the size of the pericardial effusion was found. The patient was hospitalized for 3 days under careful control for pacing. One week later, follow-up echocardiography revealed complete resolution. Pacemaker interrogation was done after 2 weeks and then 1 month later, which showed acceptable electrical parameters of both leads (pacing threshold, 0.5 V; pulse width, 0.4 ms).

### Case 2

**Figure 2:**
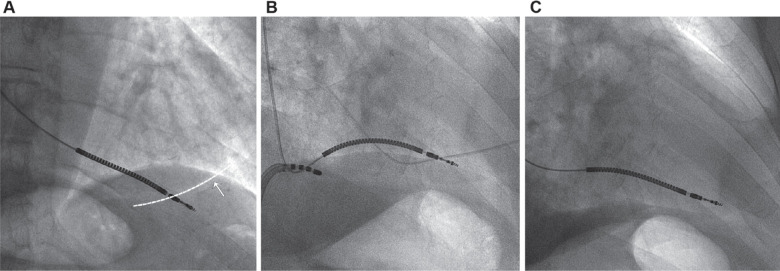
Chest fluoroscopy of patient 2. **A:** The right ventricular lead tip is in the pericardial space, and a mild pericardial effusion is visible (arrow). The border of the heart is marked with a dashed line. **B:** Retracting the displaced lead with an ablating catheter. **C:** The lead is in its proper place.

A 77-year-old man presented to our department with clinical ventricular tachycardia attacks (rate, 300 bpm) and loss of consciousness. He had a history of dilated cardiomyopathy, chronic kidney disease, and hypertension. The transthoracic echocardiography showed global hypokinesia, an ejection fraction of 30%–35%, and mild mitral regurgitation. The patient received a single-chamber ICD and was discharged in good condition. One week later, loss of capture was observed during pacing analysis. During fluoroscopy, perforation of the RV and protrusion of the tip of the active fixation RV lead (Durata™ 7120; Abbott) into the pericardial space were visible **(Figure 2A)**. Because of an increased risk of infection in this patient and reluctance to undergo surgery, he was then transferred to the catheterization laboratory of our center and prepared for the intervention. Using an Agilis sheath and an ablation catheter (OSYPKA MEDICAL GmbH, Rheinfelden, Germany), we made a hook and pulled the atrial part of the ICD lead backward with the same technique used in case 1 **(Figure 2B)**. Pacing and sensing were subsequently normalized, and the RV wave was 10 mV. We then withdrew the sheath and ablation catheter **(Figure 2C)**. The patient was discharged in good condition after 3 days of close control.

Post-procedural echocardiography showed no pericardial effusion. Within 2 years of follow-up, the patient had good pacing and sensing parameters and did not develop any complications.

### Case 3

**Figure 3:**
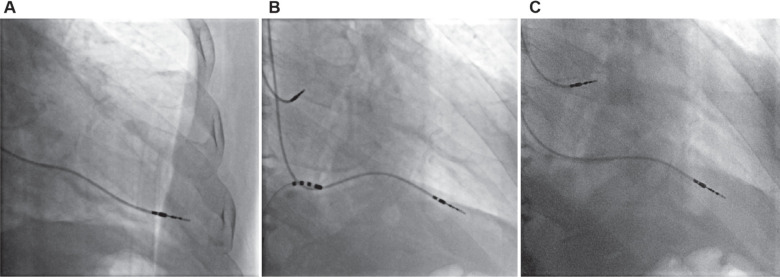
Chest fluoroscopy of patient 3. **A:** The right ventricular lead tip is in the pericardial space. **B:** Retracting the displaced lead with an ablating catheter. **C:** The lead is in its proper place.

Our third case was an 81-year-old man with tachycardia– bradycardia syndrome and a history of hypertension, dyslipidemia, cerebrovascular accident, and percutaneous transluminal angioplasty of the carotid artery. The patient received a dual-chamber pacemaker in our center, and the implantation was eventless. The post-implantation pacing analysis showed an atrial pacing threshold of 0.25 V, a pulse duration of 0.4 ms, a ventricular pacing threshold of 0.5 V, and a pulse duration of 0.4 ms. Two weeks after discharge, we noticed an increased threshold (5.5 V) in the active fixation ventricular lead (CapSureFix Novus 5076; Medtronic) during pacing analysis. During fluoroscopy, we noticed the RV lead protrusion into the pericardial space **(Figure 3A)**. Because of the patient’s condition and not giving consent for surgical repositioning of the lead, we preferred to try percutaneous transcatheter lead repositioning. Using a CryoCath sheath (Medtronic) and an ablating catheter and similar to our previous cases, we repositioned the RV lead in the RV apex **(Figure 3B)**. The ventricular threshold was then reduced to 1 V, and the patient was discharged in good condition after 3 days **(Figure 3C)**. Two weeks later, the patient had normal pacing parameters (pacing threshold, 1 V; pulse width, 0.4 ms) with no change in pacing parameters during serial follow-ups.

## Discussion

In these 3 cases, we achieved successful percutaneous transcatheter retraction and repositioning of RV pacing leads in patients with dislodged ventricular leads. This method can be used in patients who refrain from undergoing open surgical intervention or have comorbidities. This alternative method can also reduce the risk of pocket infection in such patients. However, it should be noted that it was not possible to withdraw the active helix without opening the device pocket in these patients, which is not part of the routine practice.

Nonetheless, touching the myocardium of the RV apex by the tip of the lead is enough for pacing (similar to passive leads). Although there is still a higher risk of perforation when the ventricular lead is placed in the apical position,^5^ the follow-up of our patients was eventless. We previously had a successful experience in repositioning a dislodged atrial pacing lead percutaneously^6^ and suppose that the present case report documents a novel method for lead repositioning. Additionally, decreasing the infection rate and reducing the patient’s pain level compared to surgical repositioning can rationalize the cost of this method. However, one should note that this procedure should be performed by an experienced electrophysiologist and in a highly qualified center to prevent and control complications.
